# Classification of Obesity Based on Weight History: Perceptions of People With Obesity

**DOI:** 10.1111/cob.70068

**Published:** 2026-01-14

**Authors:** Bruno Halpern, Simone van de Sande‐Lee, Maria Edna de Melo, Rodrigo N. Lamounier, Cintia Cercato, Paulo Augusto Carvalho Miranda, Rodrigo O. Moreira, Mario Kehdi Carra, Cesar Luiz Boguszewski, Marcio C. Mancini

**Affiliations:** ^1^ Obesity Center Nove de Julho Hospital São Paulo Brazil; ^2^ Department of Internal Medicine Federal University of Santa Catarina (UFSC) Florianópolis Brazil; ^3^ Obesity Unit, Department of Endocrinology, Hospital das Clínicas University of São Paulo São Paulo Brazil; ^4^ Federal University of Minas Gerais (UFMG) Belo Horizonte Brazil; ^5^ Santa Casa de Belo Horizonte Belo Horizonte Brazil; ^6^ Endocrinology Department at Mater Dei Health System Belo Horizonte Brazil; ^7^ Instituto Estadual de Diabetes e Endocrinologia Luis Capriglione (IEDE) Rio de Janeiro Brazil; ^8^ Faculdade de Medicina de Valença Centro Universitário de Valença (UNIFAA) Valença Brazil; ^9^ Faculdade de Medicina Do Centro Universitário Presidente Antônio Carlos (FAME/UNIPAC) Juiz de Fora Brazil; ^10^ Department of Endocrinology, Hospital das Clínicas University of São Paulo São Paulo Brazil; ^11^ Endocrine Division (SEMPR), Department of Internal Medicine Federal University of Parana Curitiba Brazil

**Keywords:** body mass index, body‐weight trajectory, health care survey, obesity management, public opinion, weight stigma

## Abstract

Two Brazilian medical societies have proposed a new classification of obesity based on the maximum weight attained in an individual's lifetime, to be used in the clinical evaluation of individuals undergoing obesity treatment. This classification, which applies to adults aged 18 to 65 years, categorises people with obesity (PwO) who lose a certain percentage of their weight as having either ‘reduced’ or ‘controlled’ obesity. While the classification aims to improve patient care, there is limited data on PwO perceptions. To explore this, a cross‐sectional online survey was conducted with 500 PwO, including an explanation and clinical case of the new classification. The survey revealed that 64% of participants had never been asked by a healthcare professional (HCP) about their maximum weight. After knowing the new classification, 82% found it useful for changing perceptions about obesity treatment; 66% felt it would encourage them to seek treatment; 63% believed it would help with treatment maintenance; and 74% indicated they would feel better achieving ‘controlled obesity,’ even if the weight loss fell short of their goals. A majority agreed that the classification could help establish realistic goals (77%) and reduce biases from HCPs (69%). Overall, PwO perceived the classification as beneficial for encouraging treatment and reducing stigma.

## Summary

- What is already known about this subject
  ○ Obesity is a chronic and recurrent disease, with efforts to lose weight often counteracted by biological mechanisms that slow down weight loss and contribute to recurrent weight gain.
  ○ Modest weight loss, particularly a reduction of 5% or more, can improve obesity‐related comorbidities and overall quality of life, with greater benefits observed for weight loss exceeding 10%.
  ○ Based on the above premises, two Brazilian medical societies—the Brazilian Association for the Study of Obesity (ABESO) and the Brazilian Society of Endocrinology and Metabolism (SBEM)—proposed a new classification of obesity based on the maximum weight attained in life. This classification designates individuals who have lost a certain percentage of their weight as having ‘reduced’ or ‘controlled’ obesity, serving as an additional tool to enhance understanding of the disease and facilitate discussions about treatment.
- What this study adds
  ○ Despite being a key information for understanding weight trajectories, maximum weight attained in life is rarely asked by healthcare professionals. In fact, 64% of individuals living with obesity reported never being asked about it, although 94% stated they were certain or almost certain of their maximum weight.
  ○ Despite evidence indicating modest weight loss improves health outcomes, 65% of individuals living with obesity believe that reaching a normal body mass index is necessary for improving their health and quality of life, highlighting that important clinical information is not reaching its target audience.
  ○ This is the first study to confirm that the ABESO/SBEM classification was perceived as a useful tool in clinical practise by individuals living with obesity, for: (1) setting realistic goals, (2) encouraging the pursuit and maintenance of obesity treatment, and (3) reducing stigma. Individuals living with obesity generally agreed that the classification should be adopted by health professionals.

## 1 Introduction

The diagnosis and classification of obesity are currently based on body mass index (BMI), although various alternative classification methods have been proposed—including, more recently, the Lancet Comission report and the European Association for the Study of Obesity (EASO) Framework [1, 2, 3]. In 2022, the Brazilian Society of Endocrinology and Metabolism (Sociedade Brasileira de Endocrinologia e Metabologia—SBEM) and the Brazilian Association for the Study of Obesity and Metabolic Syndrome (Associação Brasileira para o Estudo da Obesidade e Síndrome Metabólica—ABESO) proposed a new evaluation method for people with obesity (PwO) based on the maximum weight attained in life (MWAL, or highest‐ever weight). Rather than serving as an alternative to BMI, or even to other newly proposed classifications, this approach is intended as an adjunct tool for both research and clinical practise, and can even be used alongside traditional and/or alternative diagnostic tools for obesity [4]. Importantly, the classification is restricted to adults aged 18 to 65 years and is based on classical BMI thresholds, without adjustment for ethnicity, as these are the thresholds currently adopted in Brazil, where the population has a predominantly mixed genetic background [5, 6]. BMI cut‐offs may need to be adjusted for specific ethnic groups in other countries or contexts.

The concept of the classification is based on two main premises: (1) obesity is a chronic and recurrent disease, where efforts to lose weight are often counteracted by the brain leading to increased hunger and reduced energy expenditure. This results in a decreased rate of weight loss (WL) during treatment and a tendency for recurrent weight gain when treatment is halted or efforts are diminished [7, 8, 9, 10, 11]; and (2) the goal of an obesity treatment is not necessarily to normalise BMI, but to improve health and quality of life. Extensive evidence indicates that modest WL, particularly a reduction of 5% or more—with even greater benefits observed for losses exceeding 10%—can improve several obesity‐related diseases. As such, this modest weight loss is recognised as a treatment target by several guidelines worldwide [4, 12, 13, 14, 15, 16, 17].

Although both premises are well established among obesity researchers and practitioners, and obesity is recognised as a chronic disease by many organisations worldwide, inquiries and descriptions about weight history remain uncommon in clinical practise, and very few publications emphasise maximum weight attained in life (MWAL) as relevant clinical information [18, 19, 20]. Many individuals still believe that effective treatment of obesity equates to normalising BMI, specifically reaching a BMI of less than 25 kg/m^2^ [21, 22]. We believe that a classification centered on weight history, aimed at setting more realistic goals, could serve as a valuable tool for disseminating both messages to a broader audience.

The classification was described in detail by Halpern et al. and it is beyond our scope to revisit the full concept here [4]. Nonetheless, its main criteria are outlined in Table 1 and are quite straightforward. Notably, it considers maximum weight attained in life (MWAL) as crucial information during medical consultations for obesity treatment, applicable in both clinical practise and research. It also acknowledges the chronic nature of obesity by considering an individual's highest lifetime BMI—excluding any weight gained during pregnancy—when diagnosing obesity. Therefore, even if a person achieves a normal BMI after weight loss, they will still be classified as having obesity, followed by the term ‘controlled’ and the corresponding percentage of weight loss achieved.

**TABLE 1:** Proposed obesity classification^a^ based on weight history [4].

Maximum BMI	Unchanged	Reduced	Controlled
30–40 kg/m^2^	< 5%	5%–9.9%	≥ 10%
40–50 kg/m^2^	< 10%	10%–14.9%	≥ 15%

Abbreviation: BMI, body mass index.

^a^ This proposed classification is based on BMI cut‐offs used for the general Brazilian population. No ethnicity‐specific thresholds have been suggested in the original document, but adaptations may be considered when applied to other populations.

Moreover, the classification conveys a positive message that weight losses exceeding 10% in individuals with an initial BMI between 30 and 40 kg/m^2^—and over 15% for those between 40 and 50 kg/m^2^—can lead to ‘controlled obesity’. Importantly, however, this classification is not intended as a guideline; it does not imply that all individuals should aim to lose more than 10%, nor does reaching the ‘controlled’ threshold mean that no further weight loss is necessary. In clinical practise, it serves as an additional tool for discussing treatment goals with patients, recognising that while weight loss is important, other measures of health and quality of life are essential for individualised target setting [4, 23, 24, 25, 26].

When publishing the document detailing the new classification, the aforementioned societies proposed conducting studies to evaluate its usefulness, including surveys with PwO, to determine their perception of the classification's potential to aid in obesity treatment and reduce stigma [4]. Consequently, this survey was applied to PwO in Brazil with two aims: (1) to assess the overall understanding of obesity as a chronic disease and the impact of WL on overall risk reduction, including awareness of MWAL as crucial information for obesity treatment, and (2) to present the new classification and gauge its perceived usefulness and validity on PwO's experiences.

## 2 Methods

A questionnaire tailored for PwO was developed by medical specialists from ABESO/SBEM and administered by the research institute IPEC (Intelligence in Research and Consulting, São Paulo, Brazil). The study design was cross‐sectional, with data collected through online surveys from February 16th to 27th, 2024. A total sample of 500 participants was defined according to internal methodological parameters developed by IPEC, based on extensive experience conducting national opinion surveys in Brazil [27, 28, 29]. Specifically, the sampling design was informed by previous large‐scale national surveys conducted by IPEC with Brazilian internet users aged 18 years or older from socioeconomic classes A, B and C (which correspond to higher and middle socioeconomic strata in Brazil, based on household assets, education level, and access to services). These prior studies, focused on health and obesity perceptions, collected self‐reported weight and height data, allowing estimation of BMI and identification of individuals with obesity (class I, II and III). Using these historical datasets, IPEC estimated the prevalence of obesity within this population and determined the feasibility of recruiting a balanced and representative sample. Quota sampling was then employed to reflect the demographic and geographic diversity of the Brazilian population with obesity and internet access, ensuring proportional representation based on variables such as age, gender, geographic region, household income, municipality type (capital vs. non‐capital) and obesity class. Participants were recruited via email, including a unique survey link and pre‐screened through a filtering process embedded in the online questionnaire. An initial question on weight and height ensured the inclusion of only those with a self‐reported BMI ≥ 30 kg/m^2^, and demographic quotas were verified before granting access to the full survey in order to maintain sampling balance. Based on a Simple Random Sampling approach, the maximum estimated margin of error was ±4 percentage points, with a 95% confidence level for the total sample.

Throughout the data collection process, IPEC applied standard validation protocols, including: (1) monitoring of duplicate IP addresses and device IDs, to avoid multiple entries from the same user or device; (2) timing analysis, assessing the total time spent on the questionnaire and the time between responses to detect inattentive or automated participation; (3) response pattern checks, flagging and excluding cases with abnormally high rates of ‘don't know,’ refusals, or repetitive/uniform answers; (4) CAPTCHA verification questions, embedded at programmed points during the questionnaire to ensure that responses were provided by human participants. Cases that did not meet these quality standards were removed and replaced in the sample.

The complete survey was conducted in Portuguese and the instrument used is detailed in Table 2.

**TABLE 2:** Survey instrument used to assess the perceptions of people with obesity about the classification of obesity based on weight history (translated).^a^

A. *Initial questions* What is your current weight? What is your height? What is your gender? What is your age? What is your level of education? Q1. Thinking about your entire life, what was the highest weight you have ever reached? Q1A. Thinking about the maximum weight you just mentioned, would you say that: It's the exact weight I've ever reached, I'm sure of it I don't know exactly if it was that, but it was very close to it It's a vague idea of the maximum weight I've ever reached I have no idea, it's what I imagine it was, a guess Q2. Has a health professional ever asked you what your maximum weight was in life? Yes No Q3. Have you ever tried to lose weight? Yes, many times Yes, a few times Yes, rarely I've never tried (SKIP TO Q8) Q4. And when you tried to lose weight, did you ever seek help from a specialised professional? Yes, every time I tried to lose weight Yes, a few times I tried to lose weight Yes, rare times I tried to lose weight I didn't seek help Q5. What happened when you tried to lose weight? (CONSIDER WHAT HAPPENED MOST OF THE TIMES YOU TRIED) I lost more weight than I expected I lost the amount of weight I expected I lost less weight than I expected I didn't lose any weight (SKIP TO Q8) Q6. Now considering the last time you tried to lose weight, what happened when you felt like you stopped losing weight? I considered the amount I lost to be good and continued with the treatment I considered the amount I lost to be good and stopped the treatment I considered the amount I lost to be small/insufficient but continued with the treatment I considered the amount I lost to be small/insufficient and stopped the treatment Q7. And which of the following situations best describes the last time you lost weight? I was able to maintain the weight I lost I regained some of the weight I lost I regained all the weight I lost I regained more weight than I had lost Q8. Which of the following statements best represents your opinion? To improve health and quality of life, a person needs to lose weight until they reach a normal BMI for their height (SKIP TO Q10) To improve health and quality of life, a person does not need to lose weight until they reach a normal BMI for their height; they just need to lose a certain percentage of their weight I don't know Q9. How much weight do you think a person needs to lose to improve their health and quality of life? Less than 5% of their weight 5% of their weight 10% of their weight 15% of their weight 20% of their weight More than 20% of their weight I don't know Q10. In your opinion, a person with obesity who loses weight and reaches a normal weight, that is, a BMI below 25 or normal for their height: Still has obesity Is cured of obesity I don't know
B. *Explanatory text* Let's talk now about a NEW proposal for classifying obesity. It is very important that you understand the explanations and examples to answer the next questions Obesity is currently classified based on the calculation of the BMI—body mass index. Many people believe that they need to lose weight until their BMI is normal. However, the body reacts every time a person tries to lose weight and the brain does everything it can to get them back to their maximum weight. The more weight you lose, the harder it is to maintain it. However, some studies show that it is possible to improve health with smaller weight losses, without needing to normalise the BMI. Based on this, a group of obesity experts proposed a new way to classify it and, with this, have a better definition for treatments. In addition to BMI, this new classification includes two main points in the evaluation of patients: the MAXIMUM WEIGHT that the person has reached IN THEIR LIFE and the AMOUNT OF WEIGHT that the person HAS ALREADY LOST. With this new classification, a person who has lost weight can be classified as having UNCHANGED OBESITY, REDUCED OBESITY or CONTROLLED OBESITY, based on the following references:**Table:** Maximum BMI Unchanged obesity Reduced obesity Controlled obesity 30–40 Less than 5% weight loss 5% to 10% weight loss More than 10% weight loss 40–50 Less than 10% weight loss 10% to 15% weight loss More than 15% weight loss The proposal also recommends that the percentage of weight loss be part of the nomenclature of the new obesity classification, as highlighted in the following example: **Table:** Maximum weight Maximum BMI Weight loss New classification 100 kg 34 (Class II obesity) 15 kg Class II obesity (15% controlled)
C. *Illustrative clinical cases* Thinking about everything we've talked about so far, let's look at the example of two people who currently weigh the same (85 kg) and have the same BMI (31), but have different weight loss trajectories: **Table:** Maria/Mario Joana/João 85 Kg/BMI 31 85 Kg/BMI 31 Same weight for the last 10 years (85 kg) Two years ago, he/she weighed 100 kg Never been treated for obesity He/she underwent treatment, lost 15 kg in the first 6 months, reached 85 kg, and maintained that weight Tests showed increased blood sugar and fat Tests improved, as did his/her health and quality of life He/she has OBESITY and can start treatment to improve his/her health According to the new classification, he/she would have CONTROLLED OBESITY (15%) Since he/she is at him/her maximum weight, it will be easier for him/her to lose weight Since he/she has already lost weight, it is harder to continue losing weight. It may be better to focus on maintaining the weight he/she has achieved If he/she loses more than 8.5 kg (10% of his/her weight), he/she would be classified as having controlled obesity If he/she is unable to lose more weight to reach the BMI goal, he/she may become discouraged and abandon treatment
D. *Final questions* Now we ask you to answer the following questions taking into account the explanations you have just read about the new classification for obesity. Q11. How useful do you believe the new classification is in changing your perception about obesity treatment? Very useful Useful Neither useful nor useless Somewhat useful Not useful at all I don't know Q12. How would the new classification make you feel if you lost less weight than you would like, but this loss was enough to be considered ‘controlled’? Much better Better Neither better nor worse Worse Much worse I don't know Q13. How much can the new classification encourage you to seek treatment for obesity? Very stimulated Stimulated Neither stimulated nor discouraged Slightly stimulated Not at all stimulated I don't know Q14. Now think about the following situation. You underwent treatment for obesity, lost weight, and now you have stopped losing. How much do you think the new classification can make you feel encouraged to continue the treatment? Very stimulated Stimulated Neither stimulated nor discouraged Slightly stimulated Not at all stimulated I don't know Q15. Finally, to what extent do you agree or disagree with the following statements? **Table:** Strongly agree Partly agree Neither agree nor disagree Partly disagree Strongly disagree Don't know The new classification could make healthcare professionals less strict with patients about weight loss 1 2 3 4 5 6 The new classification could make patients and healthcare professionals have more realistic weight loss goals 1 2 3 4 5 6 The new classification could reduce the prejudice that healthcare professionals have towards people with obesity 1 2 3 4 5 6 Healthcare professionals should adopt the new classification when treating obesity 1 2 3 4 5 6

Abbreviations: BMI, body mass index; Q, question.

^a^ The complete survey was conducted in Portuguese.

The questionnaire was divided in two distinct stages. In the first stage, people with obesity were asked questions aimed at characterising the sample and assessing their previous experiences and perceptions regarding obesity and its treatment (Table 2A).

In the second stage, participants were introduced to the new classification through a brief explanatory text (Table 2B), and clinical scenarios (a male example for men, and a female example for women) (Table 2C). Importantly, all terminology related to the classification was carefully phrased in neutral language to avoid biassing responses. After the classification presentation, participants answered additional questions to evaluate their overall perception of the proposal and its usefulness for individuals with obesity (Table 2D).

Survey results were reported as absolute numbers and percentages considering the total sample and categorised by gender, educational level, social class and obesity class based on current BMI. All data were self‐reported. Statistical analysis was performed using IBM SPSS Statistics for Windows, version 30.0 (IBM Corp., Armonk, New York, USA). Differences between groups were assessed using the chi‐square test, with statistical significance set at *p* < 0.05 using a two‐tailed test. As the analyses were exploratory in nature, no formal adjustment for multiple comparisons was applied.

In accordance with Resolution No. 510/2016 of the Brazilian National Health Council, public opinion research involving unidentified participants is exempt from review by a research ethics committee. IPEC adheres to the ethical standards of the Brazilian Association of Research Companies (ABEP) and the European Society for Opinion and Marketing Research (ESOMAR). Its procedures comply with the international quality standard for Market Research and Opinion Research ISO 20.252 and the international quality management standard ISO 9001.

## 3 Results

**FIGURE 1:**
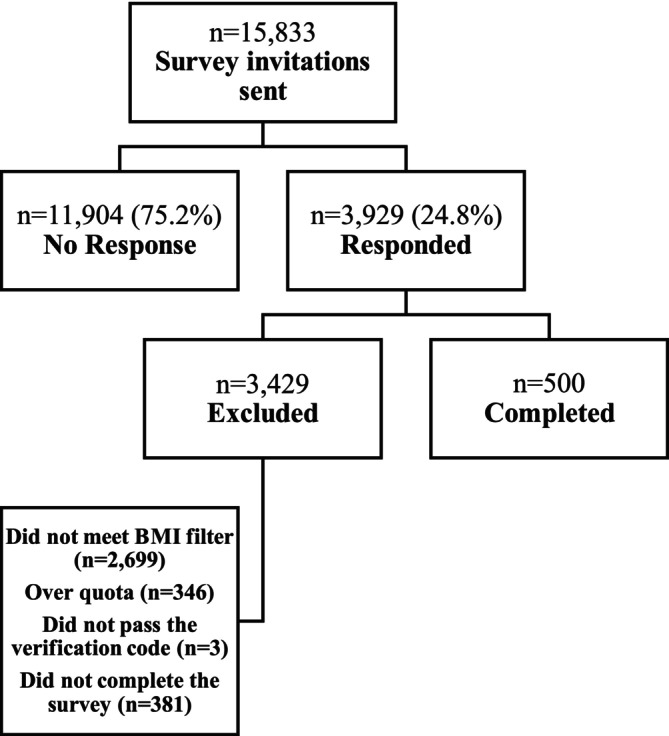
Sample disposition. BMI, body mass index.

A total of 15 833 individuals were invited to participate via email. Of these, 3929 accessed the survey link, resulting in a response rate of 24.8%. The survey was completed by 500 PwO. Sample disposition is shown in Figure 1, while the characteristics of the final sample are presented in Table 3. Complete survey results based on the total sample and divided into groups by gender, educational level, social class, and obesity class are displayed in Table S1, available in the Supporting Information.

**TABLE 3:** Sample characteristics.

	*n* (%) (total = 500 PwO)
Gender	
Male	215 (43.0)
Female	285 (57.0)
Age group	
18–34 years	140 (28.0)
35–44 years	130 (26.0)
45–59 years	163 (32.6)
≥ 60 years	67 (13.4)
Geographic region	
North/Midwest	62 (12.4)
Northeast	73 (14.6)
Southeast	262 (52.4)
South	103 (20.6)
Education level	
Elementary/high school	284 (56.8)
Higher education	216 (43.2)
Social class	
A/B	150 (30.0)
C	350 (70.0)
Municipality status	
Capital	211 (42.2)
Non‐capital	289 (57.8)
Obesity classification (based on current BMI)	
Class I	312 (62.4)
Class II	121 (24.2)
Class III	67 (13.4)

Abbreviations: BMI, body mass index; PwO, people living with obesity.

**FIGURE 2:**
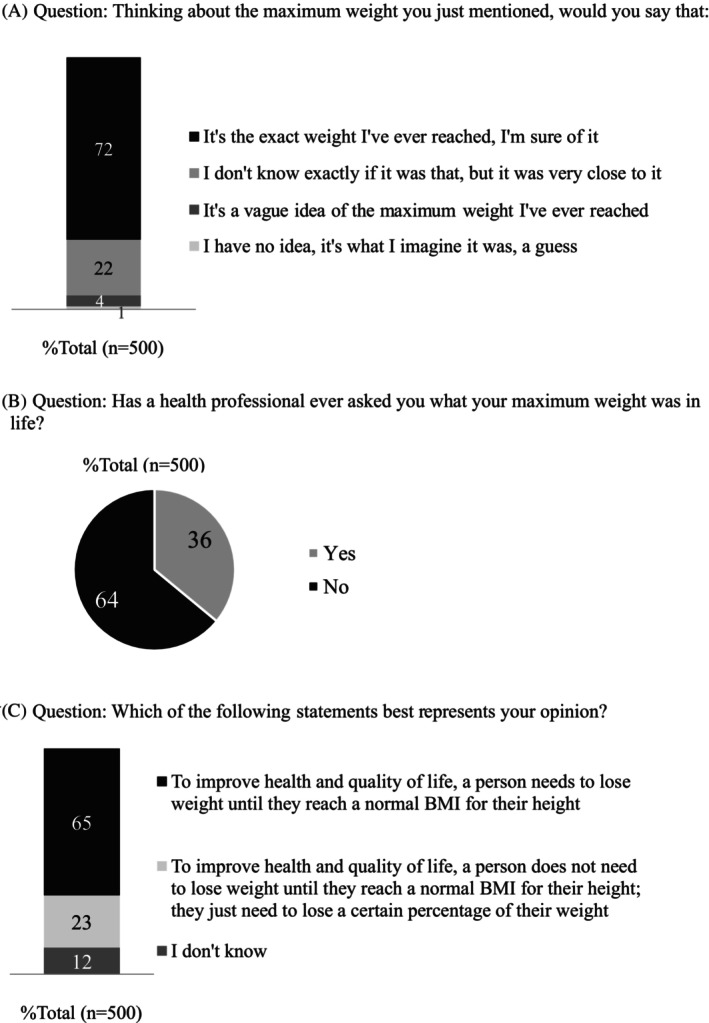
Profile of responses of people with obesity to some of the questions asked before the presentation of the concept of the new obesity classification.

Seventy‐two percent (*n* = 362/500) of respondents reported being certain about their MWAL, while 22% (*n* = 110/500) indicated they were unsure but close to the reported value. Only 4% (*n* = 22/500) stated they had a ‘vague idea’, and 1% (*n* = 6/500) had no idea (Figure 2A). Despite being certain about their maximum weight, 64% (*n* = 321/500) had never been asked about their MWAL by healthcare professionals (HCPs) (Figure 2B). This proportion was significantly higher among those with class I obesity (70%, *n* = 217/312) compared to those with class II (58%, *n* = 70/121) and III (51%, *n* = 34/67) obesity (*p* = 0.004).

A total of 95% (*n* = 473/500) of PwO reported having attempted weight loss in the past; 61% (*n* = 304/500) had tried multiple times. The proportion of respondents who had attempted weight loss many times was significantly higher among women (71%, *n* = 202/285) than men (47%, *n* = 102/215) (*p* < 0.001) and also in the class III (72%, *n* = 48/67) and II (70%, *n* = 85/121) obesity groups, compared to the class I obesity group (55%, *n* = 171/312) (*p* = 0.022). Of the total sample, 24% (*n* = 121/500) reported attempting weight loss a few times, 10% (*n* = 48/500) rarely, and 5% (*n* = 27/500) never.

Among those who attempted to lose weight, 61% (*n* = 286/473) reported seeking help from a HCP in at least one of their attempts. The percentages were higher among women (68%, *n* = 189/276, vs. 49%, *n* = 97/197 of men, *p* < 0.001), individuals with higher education (71%, *n* = 147/206, vs. 52%, *n* = 139/267 of those with elementary or high school, *p* < 0.001), and among those belonging to social classes A and B, that is, higher socioeconomic status, typically associated with lower levels of material deprivation (76%, *n* = 108/143, vs. 54%, *n* = 178/330 of those belonging to class C, *p* < 0.001). Additionally, the proportion of participants who sought professional help was greater among those with current class III (79%, *n* = 50/63) and II (70%, *n* = 80/115) obesity compared to those with class I obesity (53%, *n* = 156/295) (*p* < 0.001).

Among participants who had attempted weight loss, 73% (*n* = 345/473) reported losing less weight than expected (15%, *n* = 69/473 lost no weight at all and 58%, *n* = 276/473 lost weight but less than anticipated). This percentage was similar across all obesity classes (*p* = 0.309). Only 21% (*n* = 99/473) reported losing the expected amount, while 6% (*n* = 29/473) stated they lost more than expected. Among men, 39% (*n* = 77/197) achieved their expected weight loss or more, while 18% (*n* = 51/276) of women achieved this result (*p* < 0.001).

Considering only the last attempt, among those who tried and lost weight, 61% (*n* = 245/404) indicated that the weight loss achieved was small or insignificant; the proportion was similar across obesity classes (*p* = 0.939), but was higher among women (70%, *n* = 156/224, vs. 49%, *n* = 89/180 of men, *p* < 0.001). Interestingly, 63% (*n* = 100/159) of those who reported achieving their desired WL interrupted treatment, very similar to the 61% (*n* = 150/245) who stopped after not achieving their desired weight.

Among participants who reported attempting to lose weight and successfully lost some, 78% (*n* = 314/404) experienced recurrent weight gain. Of these, 32% (*n* = 129/404) regained all or more of the weight lost. No statistical difference was found in any of the comparisons between groups.

For 65% (*n* = 325/500) of PwO, a person would need to lose weight to reach a normal BMI to improve health and quality of life (Figure 2C). Only 23% (*n* = 116/500) responded that this would not be necessary and 12% (*n* = 59/500) were unsure. Those who indicated that normalising BMI was not necessary, or were unsure about it, were asked what percentage of weight they believed one should lose to improve health and quality of life: 1% (*n* = 1/175) said it would be less than 5%; 2% (*n* = 4/175) indicated 5%; 8% (*n* = 14/175) believed it would be 10%; 16% (*n* = 28/175) said 15%; 19% (*n* = 34/175) responded 20%; 19% (*n* = 34/175) thought more than 20% would be needed; and 34% (*n* = 60/175) did not know.

Additionally, 28% (*n* = 139/500) of PwO believe that a person who has obesity but reaches a normal BMI still has obesity, and 37% (*n* = 185/500) think that the person is cured upon reaching a normal BMI. The remaining 35% (*n* = 176/500) are unsure.

**FIGURE 3:**
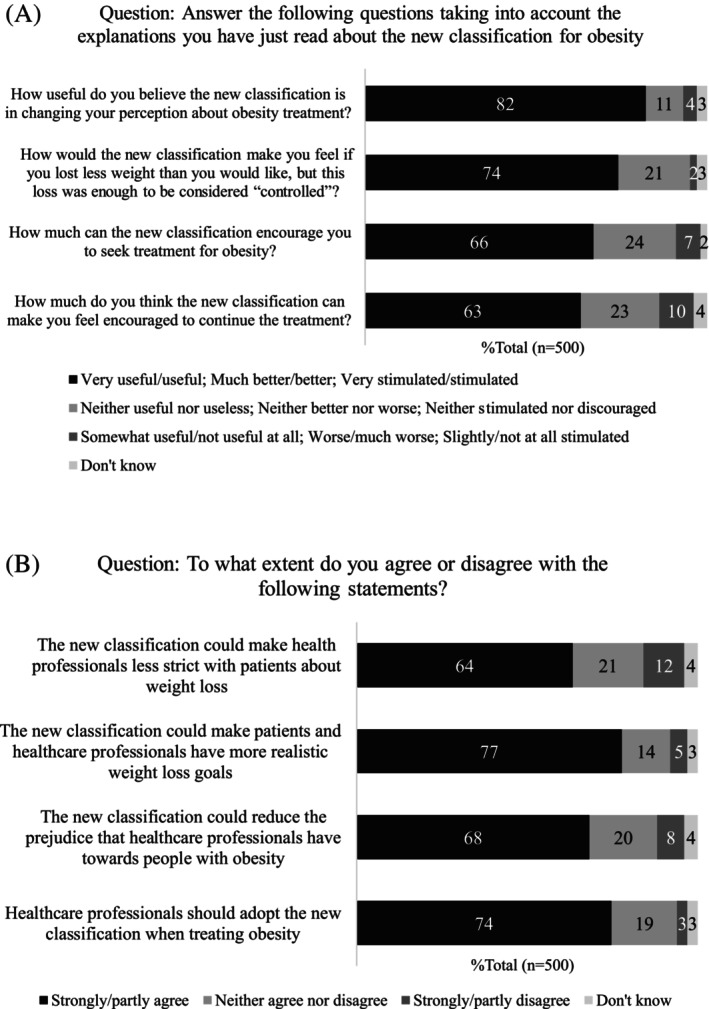
Perceptions of people with obesity about the new classification of obesity based on weight history.

In the second stage of the questionnaire, after reading the explanatory text (Table 2B) and illustrative clinical cases (Table 2C) of the proposed new obesity classification, participants answered questions regarding their perceptions of the classification (Table 2D). Of the total sample, 82% (*n* = 411/500) considered the new classification very useful or useful in changing their perspective on obesity treatment; 11% (*n* = 56/500) viewed it as neither useful nor useless; 4% (*n* = 19/500) found it somewhat useful or not useful at all; and 3% (*n* = 14/500) were unsure. The new classification would make 74% (*n* = 368/500) of respondents feel better if they lost less weight than desired, provided that the loss was sufficient for the obesity to be considered ‘controlled’. Meanwhile, 21% (*n* = 107/500) would feel neither better nor worse; 2% (*n* = 12/500) would feel worse or much worse; 3% (*n* = 13/500) didn't know. Additionally, 66% (*n* = 332/500) indicated that the new classification would encourage them to seek treatment for obesity (24%, *n* = 121/500 felt neither stimulated nor discouraged; 7%, *n* = 37/500 slightly stimulated or not at all; 2%, *n* = 10/500 were unsure) and 63%, (*n* = 318/500) reported it would motivate them to maintain treatment (23%, *n* = 114/500 felt neither stimulated nor discouraged; 10%, *n* = 49/500 slightly stimulated or not at all stimulated; 4%, *n* = 19/500 were unsure) (Figure 3A).

Finally, PwO were asked to what extent they agreed or disagreed with four statements about the new classification: 77% (*n* = 387/500) responded that they strongly or partially agreed that the new classification could help patients and HCPs set more realistic weight loss goals; 68% (*n* = 341/500) believed it could reduce the prejudice that HCPs have towards PwO; 64% (*n* = 321/500) felt it could make HCPs less strict with patients about weight loss; and 74% (*n* = 372/500) stated that HCPs should adopt the new classification in treating obesity (Figure 3B). Agreement with the statements was generally greater among women, as shown in Table S1.

## 4 Discussion

This survey offers valuable insights into the perceptions of PwO in Brazil regarding the two main concepts underpinning the proposed classification [4]. First of all, it shows that the vast majority of PwO are quite confident about their MWAL (94% when combining those who were certain with those who were uncertain but almost confident). This finding is important as one criticism of using MWAL as a core information for the classification is that it is a self‐reported measure, which raises questions about its reliability as a basis for clinical discussions. Despite this confidence and the established understanding of obesity as a chronic disease among professionals [19, 30], most patients reported never being asked about it. This information is crucial, not only for the proposed classification, but also for informing treatment and interpreting outcomes. It is anticipated that individuals whose weight is significantly lower than the MWAL will lose less weight than those close to their MWAL, a trend that has been indirectly supported by some studies [7, 8, 31, 32, 33]. At the same time, without inquiring about the MWAL, HCPs may recommend further weight reduction and establish unrealistic goals. Consequently, failing to meet these goals can be perceived as non‐compliance with treatment rather than an anticipated biologic response following significant WL. However, as this is a self‐reported questionnaire, we cannot rule out the possibility that, despite participants' confidence in their responses, some individuals may be unaware of their actual MWAL.

Interestingly, as has been shown in other surveys [34, 35], the vast majority of people with obesity have attempted to lose weight (95%), and 61% have discussed their weight at least once with a specialised HCP. Also unsurprising is the higher proportion of women and individuals from higher socioeconomic strata who reported trying to lose weight, a trend consistently observed in the literature across multiple countries [36, 37, 38]. One critical finding from this survey, however, is that the widespread understanding—at least, in scientific literature—that modest weight reductions can lower overall risks and enhance health and quality of life [13, 14, 16, 39], is not reaching those who matter most, namely, PwO. More than 60% believe that to improve their health they need to achieve a BMI lower than 25 kg/m^2^. This belief could certainly lead to feelings of failure after reaching a plateau well above this threshold, contributing to treatment discontinuation [40, 41]. Regarding the percentage of WL needed to improve health, only 11% and 27%, believe that a WL of up to 10% or 15% can lead to health improvements; thus, the majority of PwO consider weight losses in this range insufficient. This finding is in line with previous research showing unrealistic expectations regarding weight loss among people living with obesity, who frequently perceive the typical clinical target of 5%–10% as disappointing [42]. The authors of this study concluded that ‘patient education and a patient‐centered strategy would seem to be an interesting approach to negotiating reasonable weight‐loss goals in obesity and overweight management’, which aligns closely with the rationale behind our proposal.

Consequently, it is not surprising that 73% of individuals who attempted to lose weight considered their WL inadequate. Recurrent weight gain is also common, as 78% report weight gain after reaching a plateau, and 32% regaining all the weight or even more.

However, intriguingly, among those who deemed their WL sufficient, only 37% continued treatment, which is strikingly similar to the 39% of those who considered their WL insufficient. These findings indicate that treatment discontinuation is not solely linked to perceptions of success or failure but is likely also tied to a lack of understanding regarding obesity as a chronic condition and the biological tendency towards recurrent weight gain [7, 8, 40]. In this context, HCPs play an important role in helping patients set realistic expectations, reinforcing the chronic nature of obesity and providing external accountability, factors that may support long‐term engagement [43]. This is underscored by the fact that only 28% of individuals believe that those with obesity who lose weight and achieve a BMI below the obesity threshold would still be diagnosed with obesity. A survey conducted in English‐speaking countries asked a similar question in a different form: whether obesity could be cured with a healthy lifestyle. In this survey that comprises a series of different questions, 80% of respondents believed obesity could be cured, and this belief was associated with higher weight stigma scores [44]. This indirectly suggests that greater public awareness of the chronic nature of obesity could contribute to stigma reduction.

Regarding this new classification proposal [4], there was a clear perception of benefit evident in the responses. As noted in the ‘Methods’ session, and in agreement between the authors and the research institute, we took great care to present the classification in neutral terms to minimise bias. After outlining its main aspects in a straightforward manner, it became clear that the classification could facilitate PwO's understanding of several key concepts in obesity management: 82% found the classification very useful/useful; 74% indicated they would feel better if they lost less weight than desired, but enough to reach the ‘controlled’ threshold; 66% expressed that it might encourage them to seek clinical treatment; and 63% stated they would feel motivated to continue long‐term treatment if they achieved these thresholds. Additionally, there was a general consensus among PwO that HCPs should utilise and discuss this classification more often to establish more realistic goals.

When the classification was proposed a few years ago, it emphasised as an adjunct tool to aid in guiding clinical treatments rather than a replacement for traditional classifications. As an example, the Lancet Comission proposes ‘clinical obesity remission’ as a potential treatment goal [2]. Emphasising the degree of weight loss alongside more objective clinical outcomes such as remission of clinical obesity, improvement in metabolic markers, or enhanced quality of life measured by specific and validated patient reported outcome measures [24] may help set more realistic treatment targets. In this context, both frameworks can be used together, highlighting the value of weight history in guiding care. Furthermore, it was also stated that it would require validation once proposed [4, 23]. Although the concept of modest weight losses leading to improvement of comorbidities is well‐established [12, 13], we believe that validation through an epidemiological study demonstrating enhanced benefits for those with ‘controlled’ obesity, regardless of current BMI, is important and is currently underway. Moreover, this research addresses whether PwO view it as a positive tool, at least in Brazil. Additional research involving HCPs will also be valuable to understand their perceptions and to determine how the new classification can assist them in discussions about obesity with patients.

This research has several strengths, as it provides insights into PwO's perceptions of important messages in obesity treatment and highlights that a key measurement, MWAL, is rarely discussed. Furthermore, the study employed a rigorous methodology to minimise bias, using quota sampling based on demographic variables to reduce sampling bias related to population structure.

However, the research does have some limitations. It relies on a self‐reported questionnaire, and participation in the survey was voluntary, which means we cannot rule out selection bias or confirm that the individuals who completed the entire questionnaire are similar to those who did not, in terms of their overall opinions on the topic, particularly given the response and completion rate. Nevertheless, those rates are consistent with other international studies using similar online survey methods [35] and have been shown to be sufficient for producing reliable estimates in studies with comparable sample sizes [45]. As this was an online survey, individuals without internet access, particularly those from lower socioeconomic strata, may have been underrepresented. To reduce total survey time and promote adherence while minimising fatigue that might lead to careless responses, the presentation of the classification was simplified. Moreover, this is a cross‐sectional study, with the inherent limitation of not being able to evaluate changes in perceptions over time. Although it was tailored for people living with obesity, there was no direct involvement of individuals with obesity in the design of the study or the questionnaire. We acknowledge that including such participation in future research could increase the robustness and relevance of the findings. At the same time, although neutral language was used when presenting the classification and formulating the questions, we cannot rule out the presence of social desirability bias, in which individuals may provide answers that they believe will be more socially acceptable or favourable than their true opinions or behaviours [46].

In conclusion, this research demonstrated several key points: (1) individuals with obesity are aware of their MWAL, even though it is rarely discussed; (2) they believe that health improvement can only be achieved by reaching a BMI near 25 kg/m^2^, despite substantial evidence that modest weight loss can enhance health; and (3) after being presented with a brief explanation of the classification and its rationale, there was a widespread perception that the introduction of the classification based on MWAL, proposed by ABESO and SBEM, would be helpful in clinical practise, for setting realistic goals, fostering understanding of obesity as a chronic disease, and improving long‐term adherence to obesity treatment. Future validation studies are now warranted to assess the clinical utility and applicability of this classification across diverse populations and healthcare settings.

## Author Contributions

All authors have participated in (a) conception and design, or analysis and interpretation of the data; (b) drafting the article or revising it critically for important intellectual content; and (c) approval of the final version.

## Conflicts of Interest

B.H. is currently the Vice‐President of Brazilian Association of Obesity (ABESO), and President‐Elect of World Obesity Federation; has been on a speakers' bureau for Novo Nordisk, Eli‐Lilly, Boehringer Ingelheim, Astra Zeneca, Merck; has received support for attending meetings and/or travel from Novo Nordisk; has participated on advisory boards for Novo Nordisk, Eli‐Lilly, Merck, Currax, Abbott Nutrition. S.S.‐L. is currently a member of the Board of Directors of the Department of Obesity of the Brazilian Society of Endocrinology and Metabolism (SBEM); has been on a speakers' bureau for Abbott, Merck, Novo Nordisk and Lilly; and has participated on advisory boards for Eli‐Lilly. M.E.M. was President of the Brazilian Association for the Study of Obesity, has been on speakers' bureaus for EMS, Novo Nordisk, Merck; has received support for attending meetings and/or travelling from Novo Nordisk; has participated on advisory boards for Brace Pharma and Merck. R.N.L. is part of the board of directors of Brazilian Association of Obesity (ABESO) and has been on a speakers' bureau for Abbott, Astra Zeneca, Boehringer Ingelheim, Eli‐Lilly, Novo Nordisk, Merck, Medtronic; has received support for attending meetings and/or travel from Abbott, Astra Zeneca, Eli Lilly, Novo Nordisk; has participated on advisory boards for Abbott, Eli‐Lilly, Medtronic, Novo Nordisk. C.C. was President of the Brazilian Association for the Study of Obesity for two terms and is currently a member of the Obesity Department of the Brazilian Society of Endocrinology and Metabolism (SBEM); has been on speakers' bureaus for Novo Nordisk, Eli‐Lilly, Merck; has received support for attending meetings and/or travelling from Novo Nordisk; has participated on advisory boards for Novo Nordisk, Eli‐Lilly, Merck, Boehringer Ingelheim, Eurofarma. P.A.C.M. is former president of the Brazilian Society of Endocrinology and Metabolism (SBEM) in 2023/2024; has been on speakers' bureaus for Novo Nordisk, Eli‐Lilly and Merck; has received support for attending meetings and/or travelling from Novo Nordisk; has participated on advisory boards for Novo Nordisk and Eli‐Lilly. R.O.M. was president of the Brazilian Society of Endocrinology and Metabolism (SBEM) in 2019/2020; has been on speakers' bureaus for Novo Nordisk, Eli‐Lilly, Boehringer Ingelheim, Merck, EMS, Libbs, Servier, AstraZeneca, and Bayer; has received support for attending meetings and/or travelling from Novo Nordisk, AstraZeneca and Bayer; has participated on advisory boards for Novo Nordisk, Bayer, AstraZeneca, and Servier. M.K.C. and C.L.B. declare no conflicts of interest. M.C.M. was President of the Brazilian Association for the Study of Obesity for two terms, and a member of the Board of Directors of the Brazilian Society of Endocrinology and Metabolism (SBEM) for two terms; currently coordinates the Department of Obesity and Metabolic Syndrome of the Brazilian Society of Diabetes and the Department of Pharmacotherapy of ABESO; has been on speakers' bureaus for Novo Nordisk, Eli‐Lilly, Boehringer Ingelheim, Merck, EMS; has received support for attending meetings and/or travelling from Novo Nordisk; has participated on advisory boards for Novo Nordisk, Eli‐Lilly, Merck, NC Pharma, Boehringer Ingelheim.

## Supporting information


**Table S1:** Survey results.

## Data Availability

The data that support the findings of this study are available from the corresponding author upon reasonable request.
