# Supplementary material for: Classification of Obesity Based on Weight History: Perceptions of People With Obesity
Source: Clin Obes. 2026 Jan 14;16(1):e70068. doi: 10.1111/cob.70068 (PMC12803873; doi:10.1111/cob.70068)
Supplement: Supplementary file 1 — Table S1: Survey results. [file COB-16-e70068-s001.pdf]

Supporting Information

Classification of obesity based on weight history: perceptions of people with obesity

Bruno Halpern[1], Simone van de Sande-Lee[2], Maria Edna de Melo[3], Rodrigo N. Lamounier[4], Cintia Cercato[3], Paulo Augusto Carvalho Miranda[5,6], Rodrigo O. Moreira[7,8,9], Mario Kehdi Carra[10], Cesar Luiz Boguszewski[11], Marcio C. Mancini[3]

Departments/institutions

1. Obesity Center, Nove de Julho Hospital, São Paulo, Brazil.
2. Department of Internal Medicine, Federal University of Santa Catarina, Florianópolis, Brazil.
3. Obesity Unit, Department of Endocrinology, Hospital das Clínicas, University of São Paulo, São Paulo, Brazil.
4. Federal University of Minas Gerais (UFMG), Belo Horizonte, Brazil.
5. Santa Casa de Belo Horizonte, Belo Horizonte, Brazil.
6. Endocrinology Departament at Mater Dei Health system, Belo Horizonte, Brazil.
7. Instituto Estadual de Diabetes e Endocrinologia Luis Capriglione (IEDE), Rio de Janeiro, Brazil.
8. Faculdade de Medicina de Valença, Centro Universitário de Valença (UNIFAA), Valença, Brazil.
9. Faculdade de Medicina do Centro Universitário Presidente Antônio Carlos (FAME/UNIPAC), Juiz de Fora, Brazil.
10. Department of Endocrinology, Hospital das Clínicas, University of São Paulo, São Paulo, Brazil.
11. Serviço de Endocrinologia e Metabologia (SEMPR), Departamento de Medicina Interna, Universidade Federal do Paraná, Curitiba, PR, Brazil.

Corresponding author

Bruno Halpern
Brazilian Association for the Study of Obesity and Metabolic Syndrome (ABESO)
R. Mato Grosso, 306 - cj. 1711, São Paulo, Brazil, 01239-040, brunohalpern@hotmail.com

Table S1 – Survey results

Q	N resp	Answer	Total n (%)	Gender n (%)		*p*-value	Education level n (%)		*p*-value	Social class n (%)		*p*-value	Obesity classification n (%)			*p*-value
				**M**	**F**		**ES/HS**	**HE**		**A/B**	**C**		**I**	**II**	**III**	
**Q1**	500	It's the exact weight I've ever reached, I'm sure of it	362 (72.4)	162 (75.3)	200 (70.2)	0.218	204 (71.8)	158 (73.1)	0.821	107 (71.3)	255 (72.9)	0.387	215 (68.9)	95 (78.5)	52 (77.5)	0.150
		I don't know exactly if it was that, but it was very close to it	110 (22.0)	46 (21.4)	64 (22.5)		65 (22.9)	45 (29.8)		36 (24.0)	74 (21.1)		78 (25.0)	20 (16.5)	12 (17.9)	
		It's a vague idea of the maximum weight I've ever reached	22 (4.4)	6 (2.8)	16 (5.6)		11 (3.9)	11 (5.1)		4 (2.7)	18 (5.1)		17 (5.4)	3 (2.5)	2 (3.0)	
		I have no idea, it's what I imagine it was, a guess	6 (1.2)	1 (0.5)	5 (1.8)		4 (1.4)	2 (0.9)		3 (2.0)	3 (0.9)		2 (0.6)	3 (2.5)	1 (1.5)	
**Q2**	500	Yes	179 (35.8)	64 (29.8)	115 (40.4)	**0.015**	102 (35.9)	77 (35.6)	0.951	58 (38.7)	121 (34.6)	0.381	95 (30.4)	51 (42.1)	33 (49.3)	**0.004**
		No	321 (64.2)	151 (70.2)	170 (59.6)		182 (64.1)	139 (64.4)		92 (61.3)	229 (65.4)		217 (69.6)	70 (57.9)	34 (50.7)	
**Q3**	500	Yes, many times	304 (60.8)	102 (47.4)	202 (70.9)	**<0.001**	174 (61.3)	130 (60.2)	0.694	91 (60.7)	213 (60.9)	0.966	171 (54.8)	85 (70.2)	48 (71.6)	**0.022**
		Yes, a few times	121 (24.2)	71 (33.0)	50 (17.5)		64 (22.5)	57 (26.4)		37 (24.7)	84 (24.0)		87 (27.9)	24 (19.8)	10 (14.9)	
		Yes, rarely	48 (9.6)	24 (11.2)	24 (8.4)		29 (10.2)	19 (8.8)		15 (10.0)	33 (9.4)		37 (11.9)	6 (5.0)	5 (7.5)	
		I've never tried	27 (5.4)	18 (8.4)	9 (3.2)		17 (6.0)	10 (4.6)		7 (4.7)	20 (5.7)		17 (5.4)	6 (5.0)	4 (6.0)	
**Q4**	473	Yes, every time I tried to lose weight	88 (18.6)	25 (12.7)	63 (22.8)	**<0.001**	38 (14.2)	50 (24.3)	**<0.001**	40 (28.0)	48 (14.5)	**<0.001**	41 (13.9)	25 (21.7)	22 (34.9)	**<0.001**
		Yes, a few times I tried to lose weight	126 (26.6)	42 (21.3)	84 (30.4)		64 (24.0)	62 (30.1)		46 (32.2)	80 (24.2)		69 (23.4)	36 (31.3)	21 (33.3)	
		Yes, rare times I tried to lose weight	72 (15.2)	30 (15.2)	42 (15.2)		37 (14.9)	35 (17.0)		22 (15.4)	50 (15.2)		46 (15.6)	19 (16.5)	7 (11.1)	
		I didn't seek help	187 (39.5)	100 (50.8)	87 (31.5)		128 (47.9)	59 (28.6)		35 (24.5)	152 (46.1)		139 (47.1)	35 (30.4)	13 (20.6)	
**Q5**	473	I lost more weight than I expected	29 (6.1)	19 (9.6)	10 (3.6)	**<0.001**	15 (5.6)	14 (6.8)	0.509	10 (7.0)	19 (5.8)	0.371	15 (5.1)	8 (7.0)	6 (9.5)	0.309
		I lost the amount of weight I expected	99 (20.9)	58 (29.4)	41 (14.9)		50 (18.7)	49 (23.8)		35 (24.5)	64 (19.4)		70 (23.7)	17 (14.8)	12 (19.0)	
		I lost less weight than I expected	276 (58.4)	103 (52.3)	173 (62.7)		162 (60.7)	114 (55.3)		82 (57.3)	194 (58.8)		166 (56.3)	71 (61.7)	39 (61.9)	
		I didn't lose any weight (SKIP TO Q8)	69 (14.6)	17 (8.6)	52 (18.8)		40 (15.0)	29 (14.1)		16 (11.2)	53 (16.1)		44 (14.9)	19 (16.5)	6 (9.5)	

**Q6**	404	I considered the amount I lost to be good and continued with the treatment	59 (14.6)	32 (17.8)	27 (12.1)	**<0.001**	39 (17.2)	20 (11.3)	**0.009**	15 (11.8)	44 (15.9)	0.107	37 (14.7)	14 (14.6)	8 (14.0)	0.939
		I considered the amount I lost to be good and stopped the treatment	100 (24.8)	59 (32.8)	41 (18.3)		43 (18.9)	57 (32.2)		40 (31.5)	60 (21.7)		62 (24.7)	23 (24.0)	15 (26.3)	
		I considered the amount I lost to be small/insufficient but continued with the treatment	95 (23.5)	36 (20.0)	59 (26.3)		52 (22.9)	43 (24.3)		24 (18.9)	71 (25.6)		55 (21.9)	27 (28.1)	13 (22.8)	
		I considered the amount I lost to be small/insufficient and stopped the treatment	150 (37.1)	53 (29.4)	97 (43.3)		93 (41.0)	57 (32.2)		48 (37.8)	102 (36.8)		97 (38.6)	32 (33.3)	21 (36.8)	
**Q7**	404	I was able to maintain the weight I lost	90 (22.3)	46 (25.6)	44 (19.6)	0.176	56 (24.7)	34 (19.2)	0.463	29 (22.8)	61 (22.0)	0.766	61 (24.3)	20 (20.8)	9 (15.8)	0.350
		I regained some of the weight I lost	185 (45.8)	86 (47.8)	99 (44.2)		98 (43.2)	87 (49.2)		59 (46.5)	126 (45.5)		117 (46.6)	44 (45.8)	24 (42.1)	
		I regained all the weight I lost	74 (18.3)	29 (16.1)	45 (20.1)		40 (17.6)	34 (19.2)		25 (19.7)	49 (17.7)		46 (18.3)	16 (16.7)	12 (21.1)	
		I regained more weight than I had lost	55 (13.6)	19 (10.6)	36 (16.1)		33 (14.5)	22 (12.4)		14 (11.0)	41 (14.8)		27 (10.8)	16 (16.7)	12 (21.1)	
**Q8**	500	To improve health and quality of life, a person needs to lose weight until they reach a normal BMI for their height (SKIP TO Q10)	325 (65.0)	139 (64.7)	186 (65.3)	0.546	187 (65.8)	138 (63.9)	0.512	94 (62.7)	231 (66.0)	0.467	215 (68.9)	70 (57.9)	40 (59.7)	0.203
		To improve health and quality of life, a person does not need to lose weight until they reach a normal BMI for their height; they just need to lose a certain percentage of their weight	116 (23.2)	47 (21.9)	69 (24.2)		61 (21.5)	55 (25.5)		40 (26.7)	76 (21.7)		63 (20.2)	34 (28.1)	19 (28.4)	
		I don't know	59 (11.8)	29 (13.5)	30 (10.5)		36 (12.7)	23 (10.6)		16 (10.7)	43 (12.3)		34 (10.9)	17 (14.0)	8 (11.9)	
**Q9**	175	Less than 5% of their weight	1 (0.6)	0 (0.0)	1 (1.0)	0.106	1 (1.0)	0 (0.0)	0.947	0 (0.0)	1 (0.8)	0.632	1 (1.0)	0 (0.0)	0 (0.0)	0.865
		5% of their weight	4 (2.3)	3 (3.9)	1 (1.0)		2 (2.1)	2 (2.6)		0 (0.0)	4 (3.4)		2 (2.1)	1 (2.0)	1 (3.7)	
		10% of their weight	14 (8.0)	5 (6.6)	9 (9.1)		7 (7.2)	7 (9.0)		5 (8.9)	9 (7.6)		7 (7.2)	5 (9,8)	2 (7.4)	
		15% of their weight	28 (16.0)	18 (23.7)	10 (10.1)		14 (14.4)	14 (17.9)		12 (21.4)	16 (13.4)		20 (20.6)	5 (9.8)	3 (11.1)	
		20% of their weight	34 (19.4)	14 (18.4)	20 (20.2)		20 (20.6)	14 (17.9)		11 (19.6)	23 (19.3)		20 (20.6)	8 (15.7)	6 (22.2)	
		More than 20% of their weight	34 (19.4)	10 (13.2)	24 (24.2)		20 (20.6)	14 (17.9)		11 (19.6)	23 (19.3)		15 (15.5)	13 (25.5)	6 (22.2)	
		I don't know	60 (34.3)	26 (34.2)	34 (34.3)		33 (34.0)	27 (34.6)		17 (30.4)	43 (36.1)		32 (33.0)	19 (37.3)	9 (33.3)	
**Q10**	500	Still has obesity	139 (27.8)	56 (26.0)	83 (29.1)	0.745	76 (26.8)	63 (29.2)	0.735	51 (34.0)	88 (25.1)	0.112	81 (26.0)	38 (31.4)	20 (29.9)	0.114
		Is cured of obesity	185 (37.0)	82 (38.1)	103 (36.1)		109 (38.4)	76 (35.2)		53 (35.3)	132 (37.7)		113 (36.2)	40 (33.1)	32 (47.8)	

		I don't know	176 (35.2)	77 (35.8)	99 (34.7)		99 (34.9)	77 (35.6)		46 (30.7)	130 (37.1)		118 (37.8)	43 (35.5)	15 (22.4)	
**Q11**	500	Very useful	224 (44.8)	84 (39.1)	140 (49.1)	0.067	126 (44.4)	98 (45.4)	0.725	62 (41.3)	162 (46.3)	0.380	135 (43.3)	58 (47.9)	31 (46.3)	0.814
		Useful	187 (37.4)	87 (40.5)	100 (35.1)		112 (39.4)	75 (34.7)		57 (38.0)	130 (37.1)		124 (39.7)	38 (31.4)	25 (37.3)	
		Neither useful nor useless	56 (11.2)	25 (11.6)	31 (10.9)		28 (9.9)	28 (13.0)		22 (14.7)	34 (9.7)		33 (10.6)	17 (14.0)	6 (9.0)	
		Somewhat useful	15 (3.0)	7 (3.3)	8 (2.8)		7 (2.5)	8 (3.7)		6 (4.0)	9 (2.6)		11 (3.5)	2 (1.7)	2 (3.0)	
		Not useful at all	4 (0.8)	4 (1.9)	0 (0.0)		2 (0.7)	2 (0.9)		1 (0.7)	3 (0.9)		2 (0.6)	1 (0.8)	1 (1.5)	
		I don't know	14 (2.8)	8 (3.7)	6 (2.1)		9 (3.2)	5 (2.3)		2 (1.3)	12 (3.4)		7 (2.2)	5 (4.1)	2 (3.0)	
**Q12**	500	Much better	149 (29.8)	55 (25.6)	94 (33.0)	0.183	82 (28.9)	67 (31.0)	0.674	44 (29.3)	105 (30.0)	0.876	84 (26.9)	39 (32.2)	26 (38.8)	0.684
		Better	219 (43.8)	97 (45.1)	122 (42.8)		128 (45.1)	91 (42.1)		64 (42.7)	155 (44.3)		144 (46.2)	51 (42.1)	24 (35.8)	
		Neither better nor worse	107 (21.4)	48 (22.3)	59 (20.7)		62 (21.8)	45 (20.8)		33 (22.0)	74 (21.1)		70 (22.4)	24 (19.8)	13 (19.4)	
		Worse	11 (2.2)	5 (2.3)	6 (2.1)		4 (1.4)	7 (3.2)		5 (3.3)	6 (1.7)		7 (2.2)	2 (1.7)	2 (3.0)	
		Much worse	1 (0.2)	1 (0.5)	0 (0.0)		1 (0.4)	0 (0.0)		0 (0.0)	1 (0.3)		1 (0.3)	0 (0.0)	0 (0.0)	
		I don't know	13 (2.6)	9 (4.2)	4 (1.4)		7 (2.5)	6 (2.8)		4 (2.7)	9 (2.6)		6 (1.9)	5 (4.1)	2 (3.0)	
**Q13**	500	Very stimulated	132 (26.4)	47 (21.9)	85 (29.8)	0.148	72 (25.4)	60 (27.8)	0.612	42 (28.0)	90 (25.7)	0.930	77 (24.7)	35 (28.9)	20 (29.9)	0.808
		Stimulated	200 (40.0)	91 (42.3)	109 (38.2)		116 (40.8)	84 (38.9)		57 (38.0)	143 (40.9)		128 (41.0)	45 (37.2)	27 (40.3)	
		Neither stimulated nor discouraged	121 (24.2)	58 (27.0)	63 (22.1)		65 (22.9)	56 (25.9)		39 (26.0)	82 (23.4)		77 (24.7)	27 (22.3)	17 (25.4)	
		Slightly stimulated	30 (6.0)	11 (5.1)	19 (6.7)		19 (6.7)	11 (5.1)		8 (5.3)	22 (6.3)		20 (6.4)	9 (7.4)	1 (1.5)	
		Not at all stimulated	7 (1.4)	5 (2.3)	2 (0.7)		4 (1.4)	3 (1.4)		2 (1.3)	5 (1.4)		5 (1.6)	2 (1.7)	0 (0.0)	
		I don't know	10 (2.0)	3 (1.4)	7 (2.5)		8 (2.8)	2 (0.9)		2 (1.3)	8 (2.3)		5 (1.6)	3 (2.5)	2 (3.0)	
**Q14**	500	Very stimulated	117 (23.4)	45 (20.9)	72 (25.3)	0.495	67 (23.6)	50 (23.1)	0.614	29 (19.3)	88 (25.1)	0.457	67 (21.5)	31 (25.6)	19 (28.4)	0.690
		Stimulated	201 (40.2)	83 (38.6)	118 (41.4)		117 (41.2)	84 (38.9)		64 (42.7)	137 (39.1)		126 (40.4)	48 (39.7)	27 (40.3)	
		Neither stimulated nor discouraged	114 (22.8)	56 (26.0)	58 (20.4)		58 (20.4)	56 (25.9)		38 (25.3)	76 (21.7)		73 (23.4)	28 (23.1)	13 (19.4)	
		Slightly stimulated	39 (7.8)	20 (9.3)	19 (6.7)		22 (7.7)	17 (7.9)		13 (8.7)	26 (7.4)		29 (9.3)	8 (6.6)	2 (3.0)	

		Not at all stimulated	10 (2.0)	4 (1.9)	6 (2.1)		7 (2.5)	3 (1.4)		1 (0.7)	9 (2.6)		7 (2.2)	1 (0.8)	2 (3.0)	
		I don't know	19 (3.8)	7 (3.3)	12 (4.2)		13 (4.6)	6 (2.8)		5 (3.3)	14 (4.0)		10 (3.2)	5 (4.1)	4 (6.0)	
**Q15 S1**	500	Strongly agree	162 (32.4)	54 (25.1)	108 (37.9)	0.090	96 (33.8)	66 (30.6)	0.278	48 (32.0)	114 (32.6)	0.453	90 (28.8)	47 (38.8)	25 (37.3)	0.104
		Partly agree	159 (31.8)	74 (34.4)	85 (29.8)		87 (30.6)	72 (33.3)		46 (30.7)	113 (32.3)		108 (34.6)	31 (25.6)	20 (29.9)	
		Neither agree nor disagree	103 (20.6)	51 (23.7)	52 (18.2)		58 (20.4)	45 (20.8)		26 (17.3)	77 (22.0)		68 (21.8)	28 (23.2)	7 (10.4)	
		Partly disagree	28 (5.6)	14 (6.5)	14 (4.9)		15 (5.3)	13 (6.0)		12 (8.0)	16 (4.6)		20 (6.4)	3 (2.5)	5 (7.3)	
		Strongly disagree	29 (5.8)	13 (6.0)	16 (5.6)		13 (4.6)	16 (7.4)		11 (7.3)	18 (5.1)		16 (5.1)	8 (6.6)	5 (7.5)	
		Don’t know	19 (3.8)	9 (4.2)	10 (3.5)		15 (5.3)	4 (1.9)		7 (4.7)	12 (3.4)		10 (3.2)	4 (3.3)	5 (7.5)	
**Q15 S2**	500	Strongly agree	256 (51.2)	94 (43.7)	162 (56.8)	**0.021**	147 (51.8)	109 (50.5)	0.205	78 (52.0)	178 (50.9)	0.452	157 (50.3)	61 (50.4)	38 (56.7)	0.933
		Partly agree	131 (26.2)	57 (26.5)	74 (26.0)		72 (25.4)	59 (27.3)		40 (26.7)	91 (26.0)		81 (26.0)	32 (26.4)	18 (26.9)	
		Neither agree nor disagree	72 (14.4)	40 (18.6)	32 (11.2)		41 (14.4)	31 (14.4)		17 (11.3)	55 (15.7)		46 (14.7)	20 (16.5)	6 (9.0)	
		Partly disagree	18 (3.6)	11 (5.1)	7 (2.5)		7 (2.5)	11 (5.1)		8 (5.3)	10 (2.9)		12 (3.8)	3 (2.5)	3 (4.5)	
		Strongly disagree	6 (1.2)	4 (1.90	2 (0.7)		3 (1.1)	3 (1.4)		3 (2.0)	3 (0.9)		5 (1.6)	1 (0.8)	0 (0.0)	
		Don’t know	17 (3.4)	9 (4.2)	8 (2.8)		14 (4.9)	3 (1.4)		4 (2.7)	13 (3.7)		11 (3.5)	4 (3.3)	2 (3.0)	
**Q15 S3**	500	Strongly agree	193 (38.6)	55 (25.6)	138 (48.4)	**<0.001**	108 (38.0)	85 (39.4)	0.189	58 (38.7)	135 (38.6)	0.933	107 (34.3)	52 (43.0)	34 (50.7)	**0.034**
		Partly agree	148 (29.6)	71 (33.0)	77 (27.0)		87 (30.6)	61 (28.2)		43 (28.7)	105 (30.0)		102 (32.7)	29 (24.0)	17 (25.4)	
		Neither agree nor disagree	100 (20.0)	51 (23.7)	49 (17.2)		53 (18.7)	47 (21.8)		32 (21.3)	68 (19.4)		67 (21.5)	28 (23.1)	5 (7.5)	
		Partly disagree	22 (4.4)	13 (6.0)	9 (3.2)		14 (4.9)	8 (3.7)		8 (5.3)	14 (4.0)		10 (3.2)	6 (5.0)	6 (9.0)	
		Strongly disagree	18 (3.6)	14 (6.5)	4 (1.4)		7 (2.5)	11 (5.1)		4 (2.7)	14 (4.0)		12 (3.8)	3 (2.5)	3 (4.5)	
		Don’t know	19 (3.8)	11 (5.1)	8 (2.8)		15 (5.3)	4 (1.9)		5 (3.3)	14 (4.0)		14 (4.5)	3 (2.5)	2 (3.0)	
**Q15 S4**	500	Strongly agree	251 (50.2)	83 (38.6)	168 (58.9)	**<0.001**	144 (50.7)	107 (49.5)	0.673	74 (49.3)	177 (50.6)	0.532	148 (47.4)	68 (56.2)	35 (52.2)	0.575
		Partly agree	121 (24.2)	56 (26.0)	65 (22.8)		68 (23.9)	53 (24.5)		43 (28.7)	78 (22.3)		84 (26.9)	22 (18.2)	15 (22.4)	

		Neither agree nor disagree	95 (19.0)	56 (26.0)	39 (13.7)		51 (18.0)	44 (20.4)		23 (15.3)	72 (20.6)		58 (18.6)	24 (19.8)	13 (19.4)	
		Partly disagree	5 (1.0)	3 (1.4)	2 (0.7)		2 (0.7)	3 (1.4)		1 (0.7)	4 (1.1)		4 (1.3)	0 (0.0)	1 (1.5)	
		Strongly disagree	12 (2.4)	8 (3.7)	4 (1.4)		7 (2.5)	5 (2.3)		3 (2.0)	9 (2.6)		8 (2.6)	4 (3.3)	0 (0.0)	
		Don't know	16 (3.2)	9 (4.2)	7 (2.5)		12 (4.2)	4 (1.9)		6 (4.0)	10 (2.9)		10 (3.2)	3 (2.5)	3 (4.5)	

Notes: Data are presented as absolute numbers and percentages within each category. *p*-values are based on the chi-square test (bold indicate significant differences between groups at the level of $p < 0.05$). Obesity classification performed according to the current body mass index calculated from self-reported data. The complete survey was conducted in Portuguese.

Abbreviations: BMI, body mass index; ES, elementary school; F, female; HE, higher education; HS, high school; M, male; N resp, number of respondents; Q, question; S, statement.

Questions description:

Q1: Thinking about the maximum weight you just mentioned, would you say that:
Q2: Has a health professional ever asked you what your maximum weight was in life?
Q3: Have you ever tried to lose weight?
Q4: And when you tried to lose weight, did you ever seek help from a specialized professional?
Q5: What happened when you tried to lose weight? (CONSIDER WHAT HAPPENED MOST OF THE TIMES YOU TRIED)
Q6: Now considering the last time you tried to lose weight, what happened when you felt like you stopped losing weight?
Q7: And which of the following situations best describes the last time you lost weight?
Q8: Which of the following statements best represents your opinion?
Q9: How much weight do you think a person needs to lose to improve their health and quality of life?
Q10: In your opinion, a person with obesity who loses weight and reaches a normal weight, i.e. a BMI below 25 or normal for their height:
Q11: How useful do you believe the new classification is in changing your perception about obesity treatment?
Q12: How would the new classification make you feel if you lost less weight than you would like, but this loss was enough to be considered "controlled"?
Q13: How much can the new classification encourage you to seek treatment for obesity?
Q14: Now think about the following situation. You underwent treatment for obesity, lost weight, and now you have stopped losing. How much do you think the new classification can make you feel encouraged to continue the treatment?
Q15: Finally, to what extent do you agree or disagree with the following statements?
S1 – The new classification could make healthcare professionals less strict with patients about weight loss.
S2 – The new classification could make patients and healthcare professionals have more realistic weight loss goals.
S3 – The new classification could reduce the prejudice that healthcare professionals have towards people with obesity.
S4 – Healthcare professionals should adopt the new classification when treating obesity.
